# Drug-induced xerostomia and hyposalivation in patients with overactive bladder: a prospective open-label observational study comparing antimuscarinics and β3-adrenoceptor agonists

**DOI:** 10.1038/s41598-025-09720-6

**Published:** 2025-07-09

**Authors:** Magdalena Ostrowska, Layla Settaf-Cherif, Jan Kiersztyn, Adam Ostrowski, Tomasz A. Drewa, Jan Adamowicz, Maciej J. Wróbel

**Affiliations:** 1 Department of Otolaryngology and Laryngological Oncology, Collegium Medicum in Bydgoszcz, Nicolaus Copernicus University in Torun, M Curie-Sklodowskiej 9, 85-094 Bydgoszcz, Poland; 2 Department of Urology, Collegium Medicum in Bydgoszcz, Nicolaus Copernicus University in Torun, M Curie-Sklodowskiej 9, 85-094 Bydgoszcz, Poland

**Keywords:** Xerostomia, Hyposalivation, Mirabegron, Solifenacin, Drug-induced, Fox, Oral manifestations, Salivary gland diseases

## Abstract

Drug-induced xerostomia significantly affects quality of life. This study evaluated subjective and objective oral dryness in 101 patients with overactive bladder (OAB) receiving solifenacin (5 or 10 mg) or mirabegron (50 mg). Fox and Xerostomia Inventory questionnaires assessed subjective xerostomia, while unstimulated salivary flow measured objective changes. Overall, salivary flow declined from 0.35 to 0.27 ml/min (− 15.1%; *p* < 0.001), with the greatest reduction in the solifenacin 10 mg group (− 19.3%; *p* < 0.001). Mirabegron had a minimal, non-significant impact (− 5.9%; *p* = 0.42). Hyposalivation prevalence rose from 12.9% pre-treatment to 25.7% post-therapy (*p* < 0.001), especially among solifenacin users. Men had higher baseline salivation (0.45 vs. 0.27 ml/min; *p* < 0.001), though both sexes experienced comparable proportional decreases. Mirabegron in multivariable analysis markedly reduced the risk of hyposalivation (OR 0.12, 95% CI 0.02–0.90), whereas hypertension increased it six-fold (OR 6.13, 95% CI 1.02–36.78); each additional 0.1 mL of baseline saliva independently decreased the odds (OR 0.32, 95% CI 0.27–0.38). These findings underscore the need for collaboration among urologists, dentists, and otolaryngologists to identify high-risk individuals early and implement preventive strategies. Adjusting OAB pharmacotherapy regimens may help optimize treatment efficacy while minimizing xerostomia’s detrimental impact on oral health and patient quality of life.

**Supplementary Information:**

The online version contains supplementary material available at 10.1038/s41598-025-09720-6.

## Introduction

Xerostomia, defined by the subjective feeling of dry mouth, is a significant problem for both oral health and patients’ quality of life^1,2^. It is distinct from hyposalivation, which is not a feeling but a clinical condition characterized by insufficient saliva production, diagnosed, e.g., with an Unstimulated Salivary Flow (USF) test, for which the cut value to diagnose hyposalivation is 0.1 ml/min^3^. Nevertheless, these two concepts (xerostomia and hyposalivation) are closely related, although the relationship between them is not fully understood.

Saliva is critical in lubricating and moistening the oral cavity, microbial homeostasis, tooth protection, digestion, and bolus formation, and many more^4^. Thus, in xerostomia with low salivary flow, sufferers might experience difficulties with eating and swallowing, halitosis, speaking, sleep disturbances, infections, compromised taste sensation, significantly higher tooth decay rates, and difficulty wearing dental prostheses^5,6^. A common cause of xerostomia is medication use^7^.

It is reported that nearly two-thirds of the most commonly prescribed medications can cause xerostomia as an adverse effect^8^. The list is extensive and includes drug classes such as β-blockers, analgesics, anticonvulsants, benzodiazepines, antidepressants, and bronchodilators, among others^5^. Significant problems of xerostomia are also reported for antimuscarinic drugs such as solifenacin, commonly used for overactive bladder (OAB) treatment^9^.

In a study conducted by Przydacz et al., the prevalence of OAB in women (mean age 60.8 years) was 39.5% and 26.8% in men. The prevalence was strongly associated with age^10^. A similar occurrence was reported by Irwin et al., where 20.6% of adults suffered from OAB^11^.

OAB, hence, is responsible for a significant number of patients suffering from and looking for help due to their oral health and their quality of life. A constant improvement in medical care brought mirabegron—a β3-adrenoceptor agonist, the alternative therapeutic option for OAB patients, with reported fewer adverse effects rates related to dryness^12^. Nevertheless, both groups of medications have advantages and disadvantages, often forcing patients to compromise between treatment effectiveness and quality of life. Fortunately for patients with OAB, treatment allows for dose modification, drug discontinuation, and resumption depending on reported symptoms and quality of life. This approach would not be possible in patients, for instance, with hypertension, where discontinuing medication upon experiencing adverse effects is not an option.

Therefore, the authors aimed to comprehensively analyze xerostomia—as a complaint reported by OAB patients and salivary flow in those groups of patients with and without antimuscarinic and β3-mimetic medications, allowing for comparison in both conditions.

## Materials and methods

### Study design

Subjective dry mouth symptoms were validated with questionnaires, whereas objective (quantitive) assessments of salivary production were applied to measure salivation. Additional demographic factors and medical data were considered; subsequently, all data were subjected to detailed analysis to assess the relationship between treatment options, salivary function, and reported complaints.

Therefore, we conducted an open-label, prospective cohort study involving 101 patients diagnosed with OAB in an outpatient urological setting by a specialist urologist. All patients met the following criteria: adult (≥ 18 years of age), diagnosis of OAB with demonstrable impact on quality of life, willingness to participate, and ability to provide written informed consent.

Participants were allocated into one of three groups based on the type and daily dosage of medication they received:

- Group 1: Patients receiving solifenacin at a dose of 5 mg
- Group 2: Patients receiving solifenacin at a dose of 10 mg
- Group 3: Patients receiving mirabegron at a dose of 50 mg

Treatment allocation was not randomized; the final choice of drug and dose was made jointly by the treating urologist and the patient after considering symptom severity, prior anticholinergic exposure, comorbidities, patient preference and the national reimbursement scheme.

The study protocol was approved by the Local Bioethics Committee (KB/205/2023), and it was conducted in accordance with the ethical standards laid out in the Declaration of Helsinki. Written informed consent was obtained from each participant prior to enrollment.

#### Assessment procedure

Standard demographic data such as age, sex, weight, and height were collected. Additionally, information regarding comorbidities, other medications, radio or chemotherapy, and smoking history was obtained. Each patient also underwent a basic otolaryngological examination to assess any abnormalities in the oral cavity and throat, including the presence of dental caries.

All measurements were conducted twice: treatment status 0: without taking the OAB drug—(control arm) and treatment status 1: while taking OAB medications; 2 weeks after initiating treatment.

A. Evaluation of xerostomia

Xerostomia was assessed using two measurement tools:

- Fox Scale: a validated questionnaire evaluating subjective feelings of dry mouth consisting of ten questions with the possible answers of “yes” or "no." A “yes” response equals one point, and a “no” response equals zero points. The maximum score is ten, indicating severe dryness and the minimum score is zero, indicating no dryness.
- Xerostomia Inventory: This includes eleven questions addressing not only dry mouth but also dryness of the eyes, skin, and nose. Patients rate their symptoms on a 5-point scale (never, hardly ever, sometimes, often, always). Scores range from 11 to 55, with lower scores indicating less severe dryness.

B. Evaluation of salivation

Saliva production was measured by an Unstimulated Salivary Flow rate test.

To minimize the influence of environmental conditions and circadian variations on salivary production, patients were scheduled at similar times of the day. Participants were instructed to refrain from smoking, eating, or rinsing their mouths for two hours before the test. Patients were asked to accumulate saliva in the oral cavity without swallowing during the test and deposit it into a container over 5 min. The collected saliva was weighed, and based on established literature, we assumed 1 g to be equivalent to 1 ml, allowing us to report results in ml/min^13^. Hyposalivation was set to be recognized as less or equal to 0.1 ml/min.

Additionally, we measured the pH of the saliva using test strips.

#### Data analysis

The statistical analysis was performed to evaluate the differences between recorded measurements and to identify predictors of hyposalivation and changes in xerostomia symptoms. Evaluated parameters and correlations included:

I. Patient demographics, including age, sex, BMI, and comorbidities, using descriptive statistics.
II. Evaluation of Xerostomia Comparison of Fox scale and Xerostomia Inventory (XI) scores before and during OAB treatment, including mean values, 95% confidence intervals, and paired t-tests for statistical significance.
III. Salivation Analysis Assessment of unstimulated salivary flow rate before and during OAB treatment, including statistical significance (paired t-tests) and percentage differences.
IV. Gender-Based Salivation Analysis Comparison of salivary flow rates between male and female patients before and during OAB treatment using independent t-tests.
V. Severity of Hyposalivation Categorization of salivary flow rates into severity levels using Ito’s classification (I < 0.033 ml/min: severe hyposalivation, II 0.033–0.066 ml/min: moderate hyposalivation, III 0.066–0.1 ml/min: mild hyposalivation, and IV > 0.1 ml/min: no hyposalivation)^14^, and analysis of changes in severity during OAB treatment. Spearman’s rank correlation was used to assess relationships with severity levels.
VI. Risk Factors for Hyposalivation Evaluation of patient characteristics and comorbidities associated with developing hyposalivation during OAB treatment using statistical significance (t-tests) and percentage differences. Relationship between salivary production, questionnaire scores, and the presence of comorbidities before and during OAB treatment using Pearson and Spearman correlation coefficients.
VII. Multivariable analysis for the prediction of post-treatment hyposalivation. Post-treatment hyposalivation was modelled with multivariable logistic regression. All clinically relevant covariates were forced into the model a priori: treatment group (reference = solifenacin 5 mg; solifenacin 10 mg; mirabegron), sex, hypertension, diabetes, rheumatologic disease, neurologic disease, lumbar-spine problems, and baseline unstimulated salivary volume. To improve clinical interpretability, baseline saliva was rescaled in 0.1 mL increments (i.e. a one-unit change in the model corresponds to an additional 0.1 mL secreted at baseline). Collinearity was examined using variance inflation factors (all < 2.5). Model discrimination was assessed by the area under the receiver-operating characteristic curve (AUC) with a bias-corrected bootstrap 95% confidence interval (2,000 resamples). Overall model fit was evaluated with the likelihood-ratio (LR) χ^2^ test and McFadden’s pseudo-R^2^.

### Statistical evaluation

Comparative analyses included the Student’s t-test for continuous variables with a normal distribution and the Mann–Whitney U test for continuous variables with a non-normal distribution. Categorical variables, such as the frequency of hyposalivation, were compared using the chi-squared test or Fisher’s exact test where appropriate. Pearson’s and Spearman’s correlation coefficients were used to assess the relationship between questionnaire scores (Fox and Xerostomia Inventory) and objective measures of saliva production. Statistical significance was set at *p* < 0.05.

The study population size was determined based on a power analysis conducted before the study. Assuming an effect size of 0.5, a power of 80%, and an alpha level of 0.05, the required sample size was calculated to ensure sufficient statistical power for detecting significant differences in subjective (questionnaire-based) and objective (saliva production) outcomes. The analyses were performed using TIBCO Software Inc. Statistica 13.3 software.

## Results

### Groups characteristics

Total number of patients was 101. A combined 80 patients were on solifenacin, with 28.7% (n = 29) on a 10 mg dose and 50.5% on a 5 mg dose (n = 51). 20.8% of the study group (n = 21) were prescribed mirabegron 50 mg.

The median age of the analyzed cohort was 67.4 years. Of the 101 patients, 60.4% were female (n = 61), and 39.6% (n = 40) were male. The median BMI for the cohort was 28.9 kg/m^2^. The median ambient temperature during the study was 22 °C.

66.3% patients (n = 67) has been diagnosed with hypertension, 22.7% (n = 23) of the population has diabetes mellitus, 16.8% (n = 17) of the cohort reported rheumatological conditions (there was no Sjogren disease in patients reports), 7.9% (n = 8) of the patients had neurological conditions (for ex. TIA, stroke history), 59.4% (n = 60) presented with lumbar spinal degeneration, 3.0% (n = 3) had a history of head and neck radiotherapy or chemotherapy, 16.8% (n = 17 patients) of the cohort were identified as nicotine users.

A detailed analysis of group characteristics is presented in Table 1, while detailed concomitant medications are presented in Table 2. Baseline xerostomia metrics—including Fox score, Xerostomia Inventory score, unstimulated salivary flow, and salivary pH—are presented in Table 3 (see the “Without drug” rows for each treatment group).

**Table 1:** Group characteristics.

	Entire study population	Solifenacin 5 mg	Solifenacin10mg	Mirabegron 50 mg
Number of patients	101	51	29	21
Median age (years)	69.0 (IQR 63.0–76.0)	69.0 (IQR 60.0–73.0) (*p* = 0.06)	71.0 (IQR 67.0–74.0) –	69.0 (IQR 64.0–76.0) (*p* = 0.38)
Women/Men (%)	60.4/39.6	51.0/49.0 (*p* = 0.12)	69.0/31.0 –	71.4/28.6 (*p* = 0.88)
Median BMI	28.0 (IQR 25.3–32.3)	28.0 (IQR 26.1–32.4) (*p* = 0.72)	29.0 (IQR 25.3–32.5) –	25.5 (IQR 24.5–32.1) (*p* = 0.21)
Number of people with hypertension (%)	66.3	56.9 (*p* = 0.29)	69.0 –	85.7 (*p* = 0.18)
Number of people with diabetes (%)	22.7	19.6 (*p* = 0.25)	31.0 –	19.0 (*p* = 0.35)
Number of people with rheumatological conditions (%)	16.8	17.6 (*p* = 0.49)	24.1 –	4.8 (*p* = 0.07)
Neurological disorders (%)	7.9	3.9 (*p* = 0.26)	10.3 –	14.3 (*p* = 0.67)
LS degeneration (%)	59.4	54.9 (*p* = 0.22)	69.0 –	57.1 (*p* = 0.40)
Post-RT/CHT head and neck (%)	3.0	3.9 (*p* = 0.28)	0.0 –	4.8 (*p* = 0.24)
Smoking (%)	16.8%	21.6 (*p* = 0.39)	13.8 –	9.5 (*p* = 0.65)
Polypharmacy (%)	40.6	35.3 (*p* = 0.41)	44.8 –	47.6 (*p* = 0.85)

LS, lumbosacral; RT, radiotherapy; CHT, chemotherapy; IQR, interquartile range.

All statistical comparisons between groups showed no significant differences relative to the solifenacin 10 mg group (*p* > 0.05).

**Table 2:** Distibution of concominant medications.

Drug group	All (n, %)	Solifenacin 5 mg (n, %)	Solifenacin 10 mg (n, %)	Mirabegron 50 mg (n, %)
Antiplatelet drugs	20 (19.8%)	8 (15.7%)	7 (22.6%)	5 (26.3%)
Oral anticoagulants	10 (9.9%)	6 (11.8%)	2 (6.5%)	2 (10.5%)
Statins and fibrates	48 (47.5%)	21 (41.2%)	16 (51.6%)	11 (57.9%)
Alpha blockers	9 (8.9%)	7 (13.7%)	1 (3.2%)	1 (5.3%)
Beta blockers	32 (31.7%)	13 (25.5%)	11 (35.5%)	8 (42.1%)
Diuretics	20 (19.8%)	9 (17.6%)	7 (22.6%)	4 (21.1%)
Metformin	18 (17.8%)	7 (13.7%)	8 (25.8%)	3 (15.8%)
Other antidiabetic drugs	7 (6.9%)	2 (3.9%)	5 (16.1%)	0 (0.0%)
ACE inhibitors or angiotensin receptor blockers	51 (50.5%)	26 (51.0%)	18 (58.1%)	7 (36.8%)
Painkillers and anti-inflammatory drugs	20 (19.8%)	12 (23.5%)	4 (12.9%)	4 (21.1%)
Allopurinol	8 (7.9%)	3 (5.9%)	4 (12.9%)	1 (5.3%)
Calcium channel blockers	21 (20.8%)	11 (21.6%)	7 (22.6%)	3 (15.8%)
Psychiatric drugs	18 (17.8%)	9 (17.6%)	5 (16.1%)	4 (21.1%)
L-thyroxine	14 (13.9%)	4 (7.8%)	6 (19.4%)	4 (21.1%)
Vitamin D3	15 (14.9%)	10 (19.6%)	5 (16.1%)	0 (0.0%)
Proton pump inhibitors	19 (18.8%)	9 (17.6%)	5 (16.1%)	5 (26.3%)
Other cardiovascular drugs	25 (24.8%)	9 (17.6%)	10 (32.3%)	6 (31.6%)

**Table 3:** Comparison of subjective and objective parameters in the study population and OAB treatment groups.

Parameter	Entire study population	Solifenacin 5 mg	Solifenacin 10 mg	Mirabegron 50 mg
Fox (points)				
Without drug	4.0 (IQR 2.00–6.00)	4.3 (IQR 2.25–6.75)	3.3 (IQR 2.00–5.00)	4.1 (IQR 2.00–6.00)
With drug	4.4 (*p* = 0.04)* (IQR 2.00–6.00)	4.4 (*p* = 0.62) (IQR 2.00–6.00)	4.7 (*p* = 0.006)* (IQR 2.00–6.00)	4.0 (*p* = 0.55) (IQR 2.00–6.00)
Change (%)	19.8 (95% CI 6.7–33.2%)*	14.3 (95% CI 2.7–31.3%)	43.4 (95% CI 6.9–79.9%)*	5.4 (95% CI − 13.7 to − 24.4%)
Xerostomia Inventory (points)				
Without drug	27.8 (IQR 21–34)	28.7 (IQR 22–35)	25.6 (IQR 20–29)	28.8 (IQR 21–33)
With drug	28.6 (*p* = 0.28) (IQR 22–34)	29.4 (*p* = 0.57) (IQR 22–36)	27.0 (*p* = 0.26) (IQR 21–32)	28.9 (*p* = 0.92) (IQR 20–33)
Change (%)	6.9 (95% CI 1.2–12.6%)	7.2 (95% CI 1.8–16.2%)	10.7 (95% CI 0.2–21.5%)	1.1 (95% CI − 7.3 to 9.5%)
Unstimulated Salivary Flow (ml/min)				
Without drug	0.35 (IQR 0.16–0.45)	0.39 (IQR 0.20–0.50)	0.30 (IQR 0.14–0.46)	0.30 (IQR 0.16–0.42)
With drug	0.27 (*p* < 0.001)* (IQR 0.10–0.40)	0.31 (*p* < 0.001)* (IQR 0.13–0.46)	0.21 (*p* < 0.001)* (IQR 0.08–0.28)	0.28 (*p* = 0.42) (IQR 0.13–0.36)
Change (%)	− 15.1 (95% CI − 26.9 to − 3.4%)*	− 16.6 (95% CI − 31.0 to − 2.2%)*	− 19.3 (95% CI − 49.9 to − 1.8%)*	− 5.9 (95% CI − 26.4 to − 14.5%)
Patients with Hyposalivation [≤ 0.1 ml/min] (%)				
Without drug	12.9	9.8	20.7	9.5
With drug	25.7 (*p* < 0.001)*	23.5 (*p* = 0.03)*	37.9 (*p* = 0.07)	14.2 (*p* = 0.31)
pH				
Without drug	6.55	6.50	6.59	6.63
With drug	6.39 (*p* = 0.16)	6.52 (*p* = 0.90)	6.16 (*p* = 0.09)	6.42 (*p* = 0.01)*

*IQR, interquartile range.

Statistically significant results with *p* ≤ 0.05.

### Baseline group comparability

Before modelling outcomes, we formally compared the three treatment groups on every baseline covariate. Continuous variables—age, Fox score, Xerostomia Inventory score, unstimulated salivary flow and resting salivary pH—were non-normal, so we used Kruskal–Wallis tests (all 3-group df = 2). Test statistics ranged from H = 1.8 to 3.0; all *p*-values were > 0.20, indicating no significant between-group differences. Categorical covariates (female sex, hypertension, diabetes, rheumatologic disease, neurologic disease, lumbar-spine problems) were examined with Pearson’s χ^2^ tests (χ^2^ = 0.27–5.64, df = 2); none reached the α = 0.05 threshold (lowest *p* = 0.060 for hypertension). To complement hypothesis testing we calculated standardized mean differences (SMDs) for each variable: all |SMD| values were < 0.25 except hypertension vs mirabegron (0.40) and age vs solifenacin 5 mg (− 0.47), confirming generally good balance and identifying those covariates most in need of adjustment in the multivariable model. The analysis is available in Supplementary Table S1.

### Xerostomia evaluation

#### Fox scale

The average Fox score for the entire population rose from 4.0 without OAB treatment to 4.4 with treatment, indicating a statistically significant increase (*p* = 0.04) and a percentage difference of 19.8% (95% CI 6.7–33.2%).

Solifenacin 5 mg does not significantly affect the severity of dry mouth symptoms, but with a higher dose, solifenacin 10 mg, the mean Fox score increased from 3.3 to 4.7, representing a statistically significant increase (*p* = 0.006) and a 43.4% rise (95% CI 6.9–79.9%).

With mirabegron 50 mg, we observed that The Fox score decreased slightly after introducing treatment from 4.1 to 4.0 but with a non-significant percentage difference of 5.4% (95% CI − 13.7–24.4%, *p* = 0.55).

#### Xerostomia Inventory

Xerostomia Inventory (XI) percentage difference shows a general trend of increased dry mouth symptoms across all three drugs, though none reached statistical significance; solifenacin 5 mg: almost no change in XI, with a 1.1% increase (95% CI − 7.3 to 9.5%), solifenacin 10 mg: a modest increase in XI of 7.2% (95% CI 1.8–16.2%), mirabegron 50 mg: a slight increase of 6.9% (95% CI 1.2–12.6%).

### Salivation analysis assessment

In the entire study population, the mean unstimulated salivary flow decreased significantly with OAB medications use from 0.35 ml/min without OAB therapy to 0.27 ml/min during therapy (*p* < 0.001). It corresponded with a − 15.1% change in saliva production (95% CI − 26.9 to − 3.4%). With solifenacin 5 mg, the mean saliva production rate decreased from 0.39 ml/min in the absence of therapy to 0.31 ml/min with therapy. This reduction reached statistical significance (*p* < 0.001), with a percentage difference in saliva production of − 16.6% (95% CI − 31.0 to − 2.2%). Similarly, with solifenacin 10 mg, the unstimulated salivary flow was reduced: the mean rate decreased from 0.30 ml/min without therapy to 0.21 ml/min with therapy. This change was also statistically significant (*p* < 0.001) and corresponded to a − 19.3% difference in saliva production (95% CI − 49.9 to − 1.8%).

In contrast, among patients receiving mirabegron 50 mg, the mean salivary flow rate without therapy was 0.30 ml/min, slightly decreasing to 0.28 ml/min with therapy. This change was not statistically significant (*p* = 0.42), with a percentage difference in saliva production of − 5.9% (95% CI − 26.4 to 14.5%).

In the overall study population, the prevalence of hyposalivation was 12.9% in the absence of OAB medications, increasing significantly to 25.7% with therapy, regardless of the type of drug (*p* < 0.001). Within the solifenacin 5 mg group, the proportion of patients with hyposalivation rose from 9.8% without treatment to 23.5% with treatment, a statistically significant increase (*p* = 0.03). In the solifenacin 10 mg group, the percentage of patients exhibiting hyposalivation increased from 20.7% without therapy to 37.9% with therapy, approaching statistical significance (*p* = 0.07). For patients receiving mirabegron 50 mg, the rate of hyposalivation was 9.5% without therapy, which increased to 14.2% with therapy; however, this change was not statistically significant (*p* = 0.31).

### Gender-based salivation analysis comparison

Regarding sex differences, baseline salivary flow rate was significantly higher in men (0.45 ml/min) compared to women (0.27 ml/min) (*p* < 0.001). During therapy, salivary flow decreased in both groups, with women exhibiting a flow rate of 0.22 ml/min and men of 0.35 ml/min (*p* = 0.02).

#### pH measurements

In the entire study population, mean saliva pH slightly decreased with therapy (6.55 to 6.39; *p* = 0.16), though results varied depending on the specific drug administered.

For solifenacin 5 mg, the change was from 6.5 to 6.52, with *p* = 0.9; for solifenacin 10 mg, the decrease was observed from 6.59 to 6.16 and did not reach scientific significance (*p* = 0.09). For patients on mirabegron 50 mg, the mean pH showed a statistically significant decrease from a median of 6.63 to 6.42 with implemented therapy (p = 0,01).

Table 3 provides a detailed presentation of both objective and subjective measurements, stratified by the entire study population and further categorized by each of the three OAB treatment regimens: solifenacin 5 mg, solifenacin 10 mg, and mirabegron 50 mg.

### Severity of hyposalivation categorization

#### Degree of salivation

Patient characteristics in relation to the observed degree of salivation during OAB treatment are presented in Table 4.

**Table 4:** Patients’ characteristics in relation to observed degree of salivation during OAB treatment.

Unstimulated salivary flow category/(ml/min)	I < 0.033	II < 0.066	III < 0.1	IV ≥ 0.1
Number of patients	8	9	3	81
Mean age (years)	71.4 ns	72.4 ns	71.7 ns	66.2
Mean BMI	29.2 ns	28.7 ns	23.3 ns	29.1
Number of patients with hypertension (%)	75.0 ns	88.9 ns	100 ns	61.7
Number of patients with diabetes (%)	37.5 ns	33.3 ns	0 ns	21.0
LS degeneration (%)	75.0 ns	88.9* *p* = 0.05	66.7 ns	54.3
Post-RT/CHT head and neck (%)	25.0* *p* < 0.001	0 ns	0 ns	1.2
At least one comorbidity [%]	100%	100%	100%	81.5%
Charlson Comorbidity Index [pts]	5.50* *p* = 0.03	5.56* *p* = 0.01	5.00 ns	3.592
Decrease in XI [pts]	6.0* *p* = 0.03	− 4.0 ns	5.0 ns	0.6
Percentage of patients with XI decreased by ≥ 6 points [%]	37.5 ns	11.1 ns	33.3 ns	22.2
Decrease in Fox [pts]	0.88 ns	0.44 ns	1.00 ns	0.42
Percentage of patients with FOX decreased by ≥ 3 points [%]	12.5 ns	11.1 ns	0.0 ns	8.6
Patients with initial hyposalivation (< 0.1 ml/min) [%]	75.0* P < 0.001	55.6* P < 0.001	33.3* P < 0.001	1.2

All statistical comparisons between groups were analyzed in relative to Group IV; *Statistically significant results with *p* ≤ 0.05.

ns, nonstatistical; BMI, body mass index; LS degeneration, lumbar spine degeneration; Post-RT/CHT, post-radiotherapy/chemotherapy; XI, xerostomia inventory.

Without OAB treatment, the distribution of patients across these categories was as follows: 3, 4, 2, and 92 patients, respectively. During OAB treatment, the distribution shifted to 8, 9, 3, and 81 patients, respectively. A change in severity by at least one level was observed in 17 patients. Among patients receiving mirabegron 50 mg, the unstimulated salivary flow worsened at least in one level in 1 of 21 cases (4.8%). For those taking solifenacin 5 mg, 9 of 51 patients (17.6%) experienced worsening, and for those on solifenacin 10 mg, 7 of 29 patients (24.1%) showed worsening.

Spearman’s rank correlation analysis revealed a negative correlation (r = − 0.31) between the number of comorbidities and the severity of hyposalivation after treatment.

Regarding sex differences, following OAB treatment, women constituted 75% of patients who experienced decreased salivary production, defined as hyposalivation (categories I–III), although statistical significance was not demonstrated (*p* = 0.2). When analyzing the degree of hyposalivation during OAB treatment, women accounted for 50.0% in category I (the most severe) (*p* = 0.69), 88.9% in category II (*p* = 0.08), and 100% in category III (*p* = 0.16).

#### Risk factors of developing hyposalivation

In the subgroup analysis, patients who initially did not present with hyposalivation were divided into two groups: those whose level of saliva production was still within reference value after the introduction of medications (n = 74) and those who developed hyposalivation (n = 14).

#### Hyposalivation vs age

Patients who did not develop hyposalivation were younger on average, although the difference was not statistically significant (mean age 65.7 vs 72.2 years, *p* = 0.07).

#### Hyposalivation vs comorbidities

Hypertension was observed in 58.1% of patients without hyposalivation, whereas all patients who developed hyposalivation had hypertension (*p* = 0.002). No other differences in clinical parameters were identified between the two groups.

Baseline Fox questionnaire scores differed significantly between the groups, with scores of 3.6 vs 5.15 points (*p* = 0.04). After introducing medications, the scores were 3.9 vs 5.5 points (*p* = 0.05).

Patients who did not develop hyposalivation were less likely to have any predefined risk factor compared to those who did (79.7% vs 100%, *p* = 0.06). This group’s observed risk factors were significantly lower (mean 1.52 vs 2.36, *p* = 0.01).

#### Correlation between salivary production and questionnaire scores depending on the presence of comorbidities

Only 15 out of 101 participants had no comorbidities. These individuals exhibited significantly lower baseline scores on the Fox and XI questionnaires before treatment: 2.47 vs. 4.25 (*p* = 0.01) for the Fox questionnaire and 23.8 vs. 28.5 (*p* = 0.05) for the XI questionnaire. After treatment, their scores were also lower, at 3.06 vs. 4.65 (*p* = 0.03) for the Fox questionnaire and 25.0 vs. 29.2 (*p* = 0.08) for the XI questionnaire, though the latter was not statistically significant. Baseline salivary production (ml/min) was higher in participants without comorbidities (0.42 vs. 0.33, *p* = 0.20). However, after treatment initiation, the difference became statistically significant, with values of 0.38 vs. 0.25 (*p* = 0.03). The change in salivary production after treatment in participants without comorbidities was from 0.42 to 0.38 (*p* = 0.63). It should be noted, however, that this group comprised only 15 individuals. Among participants with comorbidities, salivary production decreased from 0.33 to 0.25, which was statistically significant (*p* = 0.02).

In Spearman’s correlation analysis, the number of comorbidities correlated with baseline Fox questionnaire scores (r = 0.29), XI questionnaire scores (r = 0.22), and baseline salivary production (r = − 0.30). After treatment, the number of comorbidities correlated with Fox questionnaire scores (r = 0.25) but not with XI questionnaire scores (r = 0.11). Salivary production after treatment showed the strongest correlation with the number of comorbidities (r = − 0.35). Additionally, the number of comorbidities was weakly negatively correlated with the percentage change in salivary production (r = − 0.20).

### Multivariable analysis for the prediction of post-treatment hyposalivation.

In the fully adjusted model (Table 5), mirabegron therapy was independently protective, reducing the odds of hyposalivation by 88% compared with solifenacin 5 mg (adjusted OR 0.12, 95% CI 0.02–0.90, *p* = 0.040). Hypertension emerged as the main risk factor (OR 6.13, 95% CI 1.02–36.78, *p* = 0.047). Each additional 0.1 mL of baseline saliva decreased the risk (OR 0.32, 95% CI 0.27–0.38, *p* < 0.001), indicating a strong dose-dependent protective effect. Solifenacin 10 mg, male sex, diabetes, rheumatologic or neurologic comorbidity, and lumbar-spine problems were not significantly associated with the outcome (*p* > 0.05 for all). The model showed excellent discrimination (AUC 0.91, 95% CI 0.84–0.96) and good overall fit (LR χ^2^ = 40.9, df = 9, *p* = 2.2 × 10^−7^; pseudo-R^2^ = 0.44).

**Table 5:** Multivariable logistic regression predicting post-treatment hyposalivation.

Predictor	β	SE	Adjusted OR (95% CI)	*P*
Solifenacin 10 mg vs 5 mg	0.303	0.741	1.35 (0.32–5.79)	0.683
Mirabegron vs Solifenacin 5 mg	− **2.103**	**1.02**	**0.12 (0.02**–**0.90)**	**0.040**
Male vs Female	− 0.001	0.786	1.00 (0.21–4.66)	0.999
Hypertension (yes vs no)	**1.813**	**0.914**	**6.13 (1.02**–**36.78)**	**0.047**
Diabetes (yes vs no)	− 0.710	0.819	0.49 (0.10–2.45)	0.386
Rheumatologic disease (yes vs no)	− 0.636	0.827	0.53 (0.10–2.68)	0.442
Neurologic disease (yes vs no)	0.455	1.164	1.58 (0.16–15.44)	0.696
Lumbar-spine problems (yes vs no)	1.299	0.777	3.66 (0.80–16.80)	0.095
Baseline salivary volume (per 0.1 mL)	− **1.136**	**0.319**	**0.32 (0.27**–**0.38)**	** < 0.001**

Odds ratios (ORs) are adjusted for all listed covariates. Reference categories are shown after “vs.”. Bold values denote statistical significance at α = 0.05. Odds ratios < 1 indicate a protective association (lower odds of hyposalivation), whereas ORs > 1 indicate increased odds. β = log-odds coefficient; SE = standard error.

## Discussion

Oral dryness as an adverse effect of antimuscarinic therapy was based primarily on a general patient-reported complaint without employing detailed scoring systems. Chapple et al., in their clinical trial assessing the safety of solifenacin and tolterodine, reported that nearly one-third of participants experienced a subjective sensation of dry mouth^15^. In our study, we used two specific assessment tools to quantify the severity of xerostomia in OAB patients more precisely. Our observations support prior evidence indicating that antimuscarinic therapy, particularly at higher doses, may substantially exacerbate dry mouth symptoms. Previous studies have reported that the solifenacin side-effect profile is dose-dependent, while lower doses generally pose less risk of xerostomia^16,17^. In our group, we observed that increasing the dose to 10 mg resulted in a marked rise in the mean Fox score from 3.3 to 4.7, corresponding to a statistically significant 43.4% increase. In contrast, mirabegron 50 mg appears to have a milder risk of adverse effects, which aligns with earlier findings that β3-adrenoceptor agonists may offer an alternative for OAB patients^18^. In the multicenter study assessing the efficacy and safety of this drug, dry mouth incidence was low (1.6% patients) and similar to placebo (2.1%)^19^. Using the Xerostomia Inventory (XI) in our study provided additional insights into the effects of OAB treatments on dry mouth symptoms. Although none of the changes reached statistical significance, a general trend toward increased xerostomia scores was observed across all three drugs. In the Korean study examining the association between solifenacin and dry mouth, as assessed by XI, the scores increased by 2.8 + − 6.9^9^.

Xerostomia might be multifactorial and does not always correlate with reduced saliva production^20–22^. At the same time, data on the objective impact of OAB medications on salivary flow is limited in the literature. This may be due to the variability in methodologies used across studies, including differences in saliva collection techniques (unstimulated vs. stimulated). Thomson’s study suggests a clinically significant decrease in salivary function, which is reflected by a drop of at least 6 points in the XI score^23^. However, in our study, we did not observe a correlation between the severity of hyposalivation and changes in XI questionnaire scores. Our findings showed that OAB medications, particularly solifenacin at both 5 mg and 10 mg doses, were associated with significant reductions in saliva production. These reductions were again dose-dependent, with greater decreases observed at higher doses of solifenacin. In contrast, mirabegron 50 mg resulted in a minimal, non-significant reduction in salivary flow. It is important to note that, despite these decreases in USF, most values remained above the threshold for diagnosing hyposalivation. After initiating treatment, the mean USF dropped to 0.31 ml/min, 0.21 ml/min, and 0.28 ml/min for solifenacin 5 mg, solifenacin 10 mg, and mirabegron 50 mg, respectively. In the proposed classification system in Table 3 with four categories reflecting the severity of hyposalivation, the observed trends further support a dose-dependent effect of solifenacin on worsening salivary flow, as a shift by at least one severity level occurred in 17 patients, most frequently in those receiving solifenacin 10 mg, followed by those on solifenacin 5 mg, while only one patient treated with mirabegron 50 mg experienced such a shift.

In our study population, the prevalence of hyposalivation without any intervention was 12.9%.

In comparison, a meta-analysis conducted by Souza Pina reported an overall prevalence of 33.37% among elderly individuals (aged ≥ 60 years)^24^. Notably, in our research, the prevalence of hyposalivation significantly increased to 25.7% after initiating OAB therapy in patients who already exhibited hyposalivation at baseline. A study on childhood cancer survivors provides important data on younger patients who underwent medical interventions (median 25.3 years after diagnosis of cancer and treated with radio or chemotherapy). In this population, xerostomia was reported in 9.4% of cases. Hyposalivation, defined at a threshold of ≤ 0.2 ml/min, was observed in 32% of patients while using the stricter definition of ≤ 0.1 ml/min; as applied in our study, the prevalence was 8.9%^25^. The available literature is limited regarding specific data on diverse populations studied, precise drug dosages, and standardized measurement methods, making it challenging to draw direct comparisons across studies.

The normal pH of saliva varies from 6 to 7.5. Decreased salivary pH could have clinical implications, such as disturbances in oral microbiota balance and an increased risk of dental caries^26^. Our findings indicate that pharmacological therapy with Beta-3 adrenergic receptor agonists and muscarinic receptor antagonists might affect saliva pH, with varying statistical significance observed. A slight, non-significant decrease in mean saliva pH was noted in the entire study population. However, in the subgroup receiving mirabegron 50 mg, the mean saliva pH significantly decreased from a median of 6.63 to 6.42 (*p* = 0.01). For solifenacin 10 mg, the decrease was more pronounced (6.59 to 6.16), although it did not reach statistical significance (*p* = 0.09). The lack of statistical significance in the solifenacin group could be attributed to sample size limitations and individual variability in drug response. Although the observed pH values remain within the clinically acceptable range for saliva, shifting towards a more acidic environment may still have severe implications for oral health. It is said that the dissolution of tooth roots can begin when the salivary pH drops slightly below 6.5, and when acidity levels dip to pH 5.5 or lower, the process accelerates, leading to enamel erosion, discoloration and an increased risk of caries^27^.

Beyond the specific effects of pharmacological agents used in OAB therapy, numerous studies have identified a strong correlation between the number and types of medications taken already by patients and the prevalence of xerostomia. For instance, in the study by Krajewski et al., the five most prevalent pharmacologic categories associated with xerostomia were antidepressants (37% of patients), gastric medications (28%), vitamin D (25%), beta-blockers (24%), and opioids (24%)^28^. These findings align with data from a large American national database of 37,959 individuals, which demonstrated that these medication classes were among the top 18 most frequently prescribed and exhibited an increasing trend in usage from 1999–2000 to 2011–2012^29^. Similarly, in our cohort, beta-blockers were prescribed to 32.7% of patients, painkillers to 19.8%, psychiatric medications to 17.8%, and vitamin D3 to 14.9%. In our study, 41.6% of participants were on polypharmacy, commonly defined as the use of five or more medications. Thomson et al. further demonstrated that xerostomia was more common in individuals taking from five to nine medications and became even more prevalent among those on ten or more^30^. These findings emphasize that not only individual drug mechanisms but also the cumulative pharmacological burden can significantly influence oral health, particularly by exacerbating xerostomia and its associated symptoms.

Building on the previous discussion about the impact of polypharmacy on xerostomia, our findings further highlight the influence of comorbidities on patient-reported outcomes. Notably, only 15 out of 101 participants in our study were free from any other diseases (except OAB). Participants with comorbid conditions consistently reported higher scores on the Fox and XI questionnaires before and after the introduction of OAB treatment. Two recent review articles by Cannon and Dourieu also emphasize the relationship between xerostomia, the number of medications, and the presence of chronic diseases^31,32^. Regarding objective measurements, our findings also demonstrated that participants with comorbidities exhibited poorer baseline salivary function, which further declined after OAB treatment. The Charlson Comorbidity Index (CCI), a widely used method for categorizing patients based on the International Classification of Diseases (ICD) diagnoses, is a validated tool for predicting mortality by classifying or weighting comorbid conditions^33^. In our study, a higher CCI was associated with increased severity of hyposalivation, suggesting that multimorbidity may also contribute to salivary dysfunction.

Despite the use of medications, gender has been identified as an independent risk factor for experiencing xerostomia in the study conducted by Niklander^34^. In our research, although women represented the majority of our study population and were overrepresented in groups with reduced salivary flow, no statistically significant differences were found. Interestingly, women consistently reported worse subjective xerostomia scores (Fox and XI) and exhibited lower salivary flow both before and during therapy. This aligns with existing evidence that women generally produce less saliva and may perceive dry mouth symptoms more intensely than men. However, since the proportional decrease in salivary flow during treatment was similar across sexes—approximately 20%, this suggests the decline in salivary function following OAB treatment was independent of gender.

Our observation that hypertension independently increases the odds of post-treatment hyposalivation is consistent with previous work. Mohiti et al. reported that sustained hypertension was linked to lower salivary pH and higher viscosity in 135 adults, suggesting microvascular and autonomic influences on gland function^35^. Similarly, in 50 hemodialysis patients López-Pintor et al. found hypertension to be a strong risk factor for xerostomia (adjusted OR 5.2, 95% CI 1.1–24.9)^36^.

Potential mechanisms include (I) sympathetic over-activity and renin–angiotensin signaling that reduce salivary acinar secretion; (II) microvascular remodeling that limits glandular perfusion; and (III) xerogenic effects of several first-line antihypertensives (e.g., thiazides, β-blockers). Nevertheless, our cohort was not randomized; treatment choice and underlying comorbid profiles may have channeled hypertensive patients toward particular drugs, leaving room for selection bias. Therefore, while the concordance with earlier studies strengthens the biological plausibility of the association, causality should be inferred cautiously and verified in controlled, prospective trials.

This study has several limitations. First, the cohort was modest (n = 101), which reduces statistical power and widens confidence intervals. Second, patients differed in age, comorbidity burden and concomitant medications, introducing potential confounding. We sought to mitigate this by analyzing each patient both before and after treatment—using individuals as their own baseline—and by applying a multivariable logistic model that adjusted for all recorded covariates. Because drug choice was determined collaboratively by clinicians and patients rather than by random allocation, unmeasured preferences—such as a pre-existing fear of dry mouth—may have influenced the selection of some individuals toward mirabegron or solifenacin 10 mg, leaving residual selection bias despite our SMD checks and multivariable adjustment. Xerostomia was evaluated only once, at two weeks; symptom trajectories and patient adaptation beyond this early time-point remain unknown, so the results should be interpreted as short-term effects that warrant confirmation in longer follow-up studies. Although we took steps to minimize bias, residual confounding cannot be entirely ruled out; therefore, our findings should be considered hypothesis-generating and confirmed in larger, randomized, or stratified studies.

## Conclusions

Given the vital role of saliva, collaboration among urologists, dentists, and otolaryngologists is essential for managing patients at risk of oral health disturbances. Early identification and preventive strategies can help mitigate drug-induced adverse effects, particularly in patients with polypharmacy and comorbidities. Alternative therapies or treatment adjustments should be considered for these high-risk individuals to minimize adverse effects, improve adherence, and enhance overall satisfaction.

Our study comprehensively analyzes xerostomia and salivation in patients undergoing OAB treatment with antimuscarinic and β3-adrenoceptor agonist medications. Solifenacin, particularly at higher doses, might significantly reduce salivary flow and exacerbate dry mouth symptoms, while mirabegron appears to have a milder effect on salivary function and presents as a potential alternative with less impact on dry mouth.

## Electronic supplementary material

Below is the link to the electronic supplementary material.

Supplementary Material 1

## Data Availability

To protect participant privacy and in accordance with European data protection regulations, we did not seek participant consent to deposit the study data in a public repository. However, the underlying data are available from the corresponding author upon reasonable request, subject to ethical and institutional guidelines.
