# Supplementary material for: Drug-induced xerostomia and hyposalivation in patients with overactive bladder: a prospective open-label observational study comparing antimuscarinics and β3-adrenoceptor agonists
Source: Sci Rep. 2025 Jul 9;15:24758. doi: 10.1038/s41598-025-09720-6 (PMC12241398; doi:10.1038/s41598-025-09720-6)
Supplement: Supplementary file 1 — Supplementary Material 1 [file 41598_2025_9720_MOESM1_ESM.docx]

Supplementary Table S1

| **Variable** | **Test** | **χ²/H** | **df** | **p-value** | **SMD vs Sol 10 mg (Sol 5 mg)** | **SMD vs Sol 10 mg (Mirabegron)** |
| --- | --- | --- | --- | --- | --- | --- |
| Age (years) | Kruskal–Wallis | 1,84 | 2 | 0.399 | -0,468 | -0,238 |
| Baseline Fox score | Kruskal–Wallis | 2,44 | 2 | 0.295 | 0,366 | 0,313 |
| Baseline XI score | Kruskal–Wallis | 3 | 2 | 0.223 | 0,343 | 0,359 |
| Unstim. saliva flow (mL/min) | Kruskal–Wallis | 2,66 | 2 | 0.264 | 0,372 | -0,016 |
| Female (%) | Pearson χ² | 3,85 | 2 | 0.146 | -0,367 | 0,054 |
| Hypertension (%) | Pearson χ² | 5,67 | 2 | 0.059 | -0,251 | 0,4 |
| Diabetes (%) | Pearson χ² | 1,58 | 2 | 0.453 | -0,263 | -0,277 |
| Rheumatology (%) | Pearson χ² | 3,32 | 2 | 0.191 | -0,16 | -0,551 |
| Neurologic (%) | Pearson χ² | 2,52 | 2 | 0.284 | -0,25 | 0,12 |
| LS-spine problems (%) | Pearson χ² | 1,57 | 2 | 0.456 | -0,29 | -0,245 |

*Kruskal-Wallis or Pearson χ² statistics, p-values, and standardised mean differences (SMDs) versus the reference group (solifenacin 10 mg); Sol 5 – Solifenacin 5mg; Sol 10- Solifenacin 10mg*
